# Short-Term Effects of Air Pollution on Respiratory and Circulatory Morbidity in Colombia 2011–2014: A Multi-City, Time-Series Analysis

**DOI:** 10.3390/ijerph15081610

**Published:** 2018-07-30

**Authors:** Laura Andrea Rodríguez-Villamizar, Néstor Yezid Rojas-Roa, Luis Camilo Blanco-Becerra, Víctor Mauricio Herrera-Galindo, Julián Alfredo Fernández-Niño

**Affiliations:** 1 Departamento de Salud Pública, Universidad Industrial de Santander, Bucaramanga 680002, Colombia; 2 Facultad de Ingeniería, Universidad Nacional de Colombia, Bogota 111931, Colombia; nyrojasr@unal.edu.co; 3 Facultad de Economía, Universidad Santo Tomás, Bogota 110231, Colombia; luis.blanco@usantotomas.edu.co; 4 Facultad de Ciencias de la Salud, Universidad Autónoma de Bucaramanga, Bucaramanga 681004, Colombia; vherrera@unab.edu.co; 5 Departamento de Salud Pública, Universidad del Norte, Barranquilla 081007, Colombia; aninoj@uninorte.edu.co

**Keywords:** air pollution, morbidity, adverse effects, epidemiology, Colombia

## Abstract

Few studies have been conducted on the effect of air pollution on morbidity in Latin America. This study analyzed the effects of air pollution on respiratory and circulatory morbidity in four major cities in Colombia. An ecological time-series analysis was conducted with pollution data from air quality monitoring networks and information on emergency department visits between 2011 and 2014. Daily 24-h averages were calculated for NO_2_, PM_10_, PM_2.5_, and SO_2_ as well as 8-h averages for CO and O_3_. Separate time-series were constructed by disease group and pollutant. Conditional negative binomial regression models were used with average population effects. Effects were calculated for the same day and were adjusted for weather conditions, age groups, and their interactions. The results showed that effects of some of the pollutants differed among the cities. For NO_2_, PM_10_, and PM_2.5_, the multi-city models showed greater and statistically significant percentage increases in emergency department visits for respiratory diseases, particularly for the 5 to 9-year-old age group. These same pollutants also significantly affected the rate of emergency department visits for circulatory diseases, especially for the group of persons over 60 years of age.

## 1. Introduction

Environmental risk factors are responsible for roughly 23% of the global burden of disease [1]. In the case of Colombia, this burden is estimated to be approximately 16% [2]. Air pollution is the environmental factor that poses one of the largest environmental health problems worldwide. It has been defined as one of three priority environmental problems in Colombia [3,4], where non-transmissible chronic diseases represent the largest portion of the burden of disease: approximately 86% of the total years of life lost, adjusted by disability [5].

Several investigations that have been conducted recently have shown that air pollution affects the development of circulatory and respiratory diseases, with increased morbidity and mortality from these causes [6,7,8]. Consequently, air pollution and its effects on health are considered to be a public health problem. Nevertheless, very few studies have been conducted in Latin America on the effects of air pollution on health, particularly with regard to morbidity [9].

Some of the studies that have been conducted in Colombia have evaluated the effect of air pollution on respiratory health with specific population samples [10,11,12]. One such study, which estimated the effect of airborne particulate matter 10 μm or less in diameter (PM_10_) on mortality at the population level for the city of Bogota, reported that exposure to high levels of PM_10_ is associated with an increase in respiratory and cardiovascular mortality, especially for persons from low socio-economic strata [13,14]. Nonetheless, none of the studies in Colombia have estimated the effects of air pollution on respiratory and circulatory morbidity in the general population.

The objective of this investigation was to determine the short-term association between air pollution and respiratory and circulatory morbidity in four of Colombia’s major capital cities.

## 2. Materials and Methods

### 2.1. Type of Study, Unit of Observation, and Population

An ecological time-series study was performed using the city as the unit of observation, with repeated observations on a daily basis. The study population included all of the residents in four major capital cities in Colombia (Bogotá, Bucaramanga, Cali, and Medellín) between 1 January 2011 and 31 December 2014. Colombia is the country located in the north of South America in the intertropical zone with a large variety of weathers and ecosystems and an estimated population of 49 million people. Bogotá, Bucaramanga, Cali, and Medellín are four of the five major cities in Colombia and account for approximately 14 million people. Bogota (D.C.), Medellin and Bucaramanga are located in the Andean region while Cali is located in the Pacific region. Due to their different altitudes, the cities experience fairly different weather conditions: whereas Bogota has an annual average temperature of 14 °C, Medellin, Bucaramanga and Cali have averages between 22 °C and 24 °C.

### 2.2. Data Sources

#### 2.2.1. Morbidity Data

Anonymous information related to emergency department visits was obtained from the Individual Records on the Provision of Services [Registros Individuales de Prestación de Servicios (RIPS, Spanish acronym)] provided by the Social Protection System [Sistema de Protección Social (SISPRO, Spanish acronym)]. Daily counts of the number of emergency department visits and emergencies with observations were obtained from the records that specified the cities of Bogota, Bucaramanga, Cali, and Medellin as the place of residence, and that had the following International Classification of Disease ICD-10 diagnoses at the time of discharge:

Diseases of the Respiratory System [respiratory infections (J00–J06; J10–J18; J20–J22), asthma (J45–J46), chronic obstructive pulmonary disease (J40–J44)]; Diseases of the Circulatory System [Cardiovascular Diseases: acute myocardial infarction/angina (I20, I21, I24), conduction disorders/ arrhythmias (I44–I45; I47–I49), heart failure (I50), and Cerebrovascular Diseases: cerebrovascular accident (I60–I69)].

The information obtained from the RIPS was disaggregated by date of visit, age groups, and sex. A national study in 32 states conducted by the Ministry of health reported that the diagnostic accuracy of RIPS data, including ED visits data was 83.4% [15]. The total population of each city was used as the “exposure population” variable, which was calculated based on national projections of the total population for each year and city [16]. Since it can be reasonably assumed that there are practically no changes in the population of a city within the same year, this adjustment is primarily for the purpose of making the counts comparable among the age groups as well as among cities, given the differences in the total size of the susceptible population.

#### 2.2.2. Pollutant and Weather Data

The information related to criteria pollutants was obtained from the air quality monitoring systems (SVCA, Spanish acronym) in the four cities, for carbon monoxide (CO), nitrogen dioxide (NO_2_), sulfur dioxide (SO_2_), ozone (O_3_), and particulate matter 10 and 2.5 microns in diameter (PM_10_ and PM_2.5_, respectively). These data correspond to validated and adjusted information from 29 SVCA stations in the four cities (13 in Bogotá, 7 in Medellín, 5 in Cali, and 4 in Bucaramanga) between 1 January 2011 and 31 December 2014. Daily 24-h averages were calculated for NO_2_, PM_10_, PM_2.5_, and SO_2_ and maximum 8-h moving averages were estimated for CO and O_3_.

Weather variables (temperature in degrees Celsius, percentage of relative humidity, and precipitation in mm) were provided by the Hydrology, Meteorology, and Environmental Studies Institute [Instituto de Hidrología, Meteorología y Estudios Ambientales (IDEAM, Spanish acronym)]. The daily concentrations of each pollutant in each city and the weather variables were obtained by calculating the average concentrations from the stations with available information for each day in the time-series.

### 2.3. Statistical Analysis

For each city, preliminary daily time-series were constructed for each disease group (respiratory, cardiovascular, and cerebrovascular) and each pollutant and weather variable. Different graphic methods were used to perform an exploratory evaluation of the trajectory and seasonality of each time-series, including partial autocorrelation and cross-correlation plots. Lastly, conditional negative binomial regression models were adjusted for time and average population effects [(Generalized Estimating Equations (GEE)] in order to estimate the effects of the concentrations of each pollutant on the rates of emergency department visits, per disease group. In each of these models, the response variable was the count of each disease (*Y*) and the offset variable was the total population of each city for that year and age (*τ*). The logarithm of the rate *λ* was then modeled as E(*Y*)/τ, as a function of the independent variable *X*, so as to interpret the e^B^ associated with each *X* as an average percentage change in the rate of visits for each disease.

A time-stratified conditional binomial regression (grouped by day, month, and year) was used to control for the seasonality of the morbidity data. Thus, the effects were estimated based on the structure of the correlation of the observations when generated for the same day of the week, month, and year [17]. In addition, negative binomial models were used instead of assuming a Poisson distribution of the response variable, given the overdispersion of graphically observed count data and that the equidispersion hypothesis was consistently rejected by both the index of dispersion test (VIT) [18] and the Bohning asymptotic test [19]. GEE models were also used in order to estimate population averages [20], since they do not contain as many distributional assumptions as mixed models and estimates of population averages are adequately interpreted when calculating ecological effects, as in the case of the work herein.

The first step involved separately adjusting the models for city and pollutant. In order to estimate potential differential effects by day-lag, the effects of the pollutant values were estimated using a distributed lag model assessing lag effects up to 10 days. We estimated pollutants’ effect by age group directly by including interactions terms among pollutants and weather variables with age group. To aid in the interpretation and subsequent usefulness of the models’ coefficients, the daily pollutant concentrations were centered to the approximate whole value corresponding to 20% of the average of the time-series, and the PM_10_ and PM_2.5_ values were centered to 10 and 5 μg/m^3^, respectively, in accordance with the convention. The coefficients were calculated as percentage changes in the rates of emergency department visits for each disease group, where 100× (e^b^ − 1) when the Beta was positive and −100 × (1 − e^b^) when it was negative. In the second step constructing a multi-city GEE model we used the variable city as fixed effect and the pollutants’ effect by age group was estimated directly by including interactions terms among pollutants and weather variables with age group. The models were evaluated based on the distribution of residuals and goodness-of-fit tests. All of the analyses were conducted using STATA 14.

## 3. Results

There was a total of 3,879,383 emergency department visits for respiratory and circulatory diseases in the four cities between 2011 and 2014. The averages of the number of visits per year were 551,243 in Bogotá, 58,798 in Bucaramanga, 130,993 in Cali, and 150,326 in Medellín. Respiratory disease represented approximately 95% of the total emergency department visits, of which respiratory infection was the most common diagnosis. The graphic pattern of the respiratory disease time-series presented a seasonal behavior. Table 1 shows the total distribution of emergency department visits by city and age group.

In the city of Bogotá, average daily pollutant concentration values were obtained for nearly all of the 1461 days in the time-series, with the exception of PM_2.5_ (7% of the series was missing data). PM_2.5_ and SO_2_ concentrations were obtained in Bucaramanga for roughly 60% of the days, and criteria pollutant concentrations were obtained in Cali for less than 60% of the series, with the exception of PM_10_ (87%). In Medellín, SO_2_ concentrations were not registered and the other pollutants that were measured were present in up to 70% of the time series, with the exception of PM_10_ and PM_2.5_, which had valid measurements in 98% of the time series.

With regard to the highest average concentrations, the highest PM_10_, PM_2.5_, and NO_2_ concentrations were registered in Bogotá and Medellín, and the highest average CO concentrations occurred in Medellín and Bucaramanga. The highest SO_2_ concentrations were found in Cali and Bucaramanga, and the highest O_3_ were registered in Cali and Medellín. Table 2 summarizes the measurements of the average daily concentrations of the criteria pollutants and the weather variables for each city.

The conditional models, which were adjusted for the weather variables in the cities, presented statistically significant differences in the magnitude and statistical significance of the percentage change in emergency department visits for the three disease groups and the pollutants. Overall, the stronger and statistically significant effects of the pollutants primarily occurred on the same day (lag 0), lag 1 and lag 7. Therefore, the models of the effects per age group were calculated separately for each city and for lag 0.

**Figure 1:**
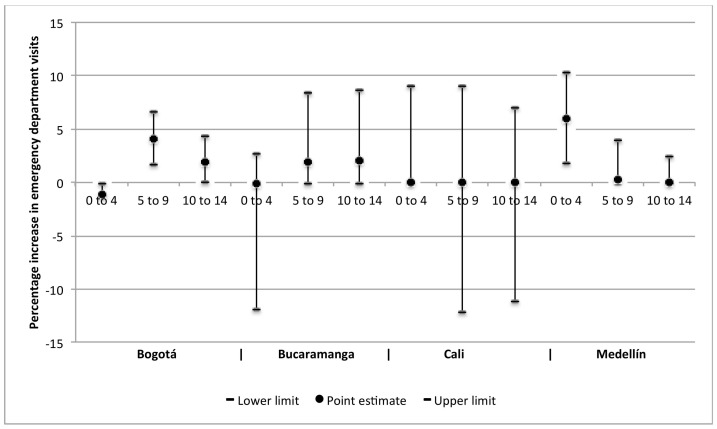
Percentage increases in emergency department visits by children for respiratory diseases associated with PM_2.5_ concentrations, per age group, in the four cities in Colombia, 2011–2014. Point estimates and 95% confidence intervals for age groups of children in years.

**Figure 2:**
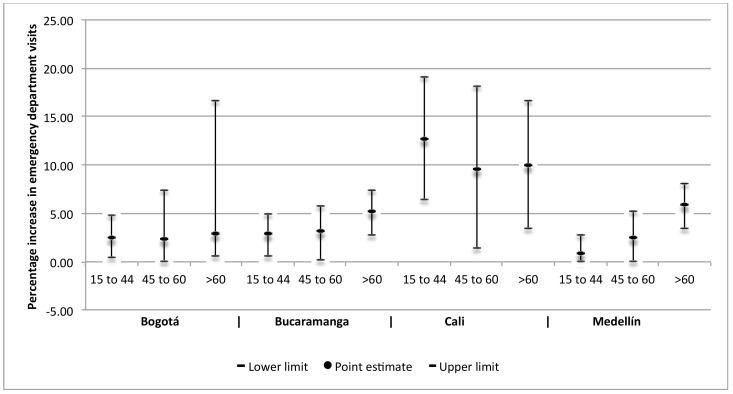
Percentage increases in emergency department visits by adults for cardiovascular diseases associated with concentrations in NO_2_, per age group, in four cities in Colombia, 2011–2014. Point estimates and 95% confidence intervals for age groups of adults in years.

In Bogotá, changes in NO_2_, SO_2_, and PM_2.5_ concentrations were related to greater and statistically significant effects on changes in emergency department visits for respiratory diseases in the group of children under 15 years of age. And changes in SO_2_, PM_10_, and PM_2.5_ were related to greater changes in emergency department visits for circulatory diseases in adults over 60 years of age. In Medellín, changes in NO_2_ and O_3_ concentrations were found to have greater and statistically significant effects on the change in visits for cerebrovascular diseases in adults, especially in those over 60 years of age, and statistically significant associations were found between PM_2.5_ and visits for respiratory diseases in children under 5 years of age. In Cali, statistically significant percentage increases were found for the effect of PM_10_ on visits for respiratory diseases in children under 5 years old, and for the effect of NO_2_ and PM_2.5_ on visits for circulatory diseases in adults. In Bucaramanga, statistically significant associations were found between SO_2_, O_3_, and PM_10_ concentrations and cerebrovascular diseases in adults, as well as between PM_10_ concentrations and cardiovascular diseases in adults, and between SO_2_ concentrations and respiratory diseases in children under 5 years old. Estimates of the effect on respiratory, cardiovascular, and cerebrovascular diseases, by type of pollutant and age group by city are presented in Supplementary material Tables S1-S4. Figure 1 and Figure 2 present the percentage increases in the emergency department visits for respiratory and cardiovascular diseases that were associated with PM_2.5_ and NO_2_, respectively, in the four cities and by age group.

Multi-city, marginal conditional models, which were fitted by including interactions terms among pollutants and weather variables with age group, indicated a stronger association between the concentrations of criteria pollutants and the percentage increases in emergency department visits for respiratory morbidities in children less than 15 years of age, particularly in the 5 to 9-year-old age group (Table 3). With regard to associations between the criteria pollutant concentrations and the percentage increase in visits for morbidities related to cardiovascular diseases in adults, a dose-response effect was seen particularly in the group over 60 years of age, and especially in relation to changes in NO_2_, PM_2.5_, and PM_10_ concentrations. For cerebrovascular diseases, associations with these same pollutants were found but only for the group of adults over 60 years of age. An association was also found between O_3_ concentrations and increased visits for respiratory diseases in children less than 5 years of age. There were few negative statistically significant associations, between air pollutants and cerebrovascular diseases, specifically in the 15–44 age group which might be explained by the low occurrence of these events in this population group probably not related to air pollution but to other congenital or individual conditions.

## 4. Discussion

The results from this study confirm the harmful, short-term effects of increased concentrations of criteria pollutants on morbidity in the general population in Colombia, measured as a percentage increase in emergency department visits for respiratory and circulatory diseases. The positive association between pollutant concentrations particularly PM_10_, PM_2.5_, SO_2_, and NO_2_ and the percentage increase in morbidities treated in emergency departments was evident even when the annual average pollutant concentrations were below the standards stipulated by Colombian legislation, and in the case of NO_2_ and SO_2_ below the World Health Organization’s (WHO) air quality guidelines [21,22]. This study also provides evidence that the effects of certain pollutants differ among cities and age groups.

Multicentric studies and meta-analyses from different regions of the world have well established an association between criteria pollutant concentrations and mortality from respiratory and cardiovascular diseases, especially in relation to PM, NO_2_, and O_3_ [23,24,25,26]. Nonetheless, very few studies of this type have been conducted in Latin America, where the Latin American health and air pollution study [(Estudio de Salud y Contaminación del Aire en Latinoamérica (ESCALA, Spanish acronym)] serves as the reference. The objective of ESCALA was to estimate the effect of daily exposure to PM_10_ and O_3_ on daily mortality from different causes in nine cities in Mexico, Brazil, and Chile [27]. That study reported an average change of 0.72% (95% CI 0.57–0.89) in the risk of death from cardiovascular diseases associated with PM_10_, and 2.44% (95% CI 1.36–3.59) from respiratory diseases such as chronic obstructive disease. For O_3_, the percentage increase in mortality from all causes was 0.16% (95% CI −0.02 to 0.33), while no significant associations were found with respiratory or circulatory diseases. In Colombia, a study by Blanco et al. [14] in collaboration with the ESCALA study group, estimated associations between PM_10_ concentrations and mortality in the city of Bogota between 1998 and 2006. They reported a 0.71% (95% CI 0.46–0.96) increase in overall mortality, a 1.43% (95% CI 0.85–2.00) increase in respiratory diseases, and a −0.03% (95% CI −0.49–0.44%) change in cardiovascular diseases, all with a lag of 3 days before death. In addition, the effects on mortality in Bogota were found to be greater among the population in the lower socioeconomic status (SES): 0.76% (95% CI 0.27–1.26) for SES 1 (low) and −0.29% (95% CI −1.16–0.57) for SES III (high) [13].

There are fewer studies on the effect of air pollution on morbidity in the general population than there are on mortality, and they are generally limited to morbidity from hospitalization [25,28]. This situation is likely due to the fact that the monitoring of vital statistics in most countries provides better-organized and centralized mortality data, while morbidity data (including treated morbidity) are not always available, especially in developing countries. When there is a need to estimate the magnitude of the acute and chronic effects of pollution, it is important to analyze the association between air pollution and morbidity, as well as the direct and indirect costs related to the health effects, not only with regard to mortality as the final effect but also in terms of early and intermediate disease stages.

A recent systematic review found 14 studies that estimated the effect of PM_2.5_ on hospital admissions for respiratory and cardiovascular diseases in North America, Europe, and southeast Asia, but did not find any in Latin America [25]. A meta-analysis of these studies estimated an overall change of 0.90 (95% CI 0.26–1.53) in cardiovascular diseases per increase in 10 µg/m^3^ of PM_2.5_ for all ages, and a change of 0.96 (95% CI −0.63 to 2.58) for respiratory diseases. It also estimated a change of 2.00% (95% 0.50–4.00) for cardiovascular diseases in adults over 65 years of age and roughly 2.1% (95% CI 0.1–4.8) for children less than 14 years of age. Although our study calculated much greater effects for the same disease groups and age ranges (adults over 65 years of age and children under 14 years old, particularly in the 5 to 9-year-old age group), direct comparisons cannot be made since the meta-analysis cited above used different interquartile range parameters and hospitalization records while our study was based on records from emergency department visits.

There are very few multi-city studies about the association between air pollution and emergency room visits for cardiovascular or respiratory diseases [29,30,31]. Chen et al. [31] performed a study in China with information from 33 hospitals and PM_10_, PM_2.5_, NO_2_, and SO_2_ measurements from 2013 to 2014. Their results, which were based on total emergency room visits, found that a 10 µg/m^3^ increase in PM_2.5_ was most strongly associated with 0 to 2-day lags [(accumulated relative risk (RR) 1.006 (95% CI 1.002, 1.009)], that NO_2_ was mostly associated with 0 to 1-day lags [RR 1.015 (95% CI 1.010, 1.019)], and that SO_2_ was more greatly associated with 0 to 2-day lags [RR 1.022 (95% CI 1.014, 1.030)]. A Canadian study by Stieb et al. [29] included information about respiratory and cardiovascular diseases between 1990 and 2000 from 7 cities and 14 hospital emergency department visits, as well as data related to all of the criteria pollutants. Their overall findings demonstrated that CO and NO_2_ concentrations on the same day (lag 0) were most consistently associated with emergency department visits for heart disease (2.1% (95% CI, 0.0–4.2%), and that ozone on lag day 2 was the pollutant that was most closely associated with visits for respiratory diseases [3.2% (95% CI, 0.3–6.2%)]. Meanwhile, a study by Alhanti et al. [30] evaluated changes in effects according to age based on information from 3 cities in the United States for time periods between 1993 and 2009, but only for emergency department visits for asthma. That study reported differential estimates for the age groups and consistently higher ones for the 5 to 8-year-old group.

Our investigation is the first in Latin America to study the association between air pollution and emergency department visits for cardiovascular and respiratory diseases based on a population perspective and a multi-city analysis. Similar to what has been reported in Canada [29], our study provides evidence of heterogeneous effects among cities. Also similar to what has been reported in Canada and China [29,31], the effects most often occur on the same day and up to day 3. These differential findings among cities can be explained by the heterogeneity of PM compositions and gas mixtures, which vary according to the transportation and industrial activities in each city. These findings may also be explained by differences in susceptibility to pollutant concentrations, which vary according to the genetic makeup and environmental factors in a particular location. Therefore, differential effects within both countries and cities need to be determined in order to inform public policy decisions related to permissible levels and the most susceptible populations, for the purpose of defining comprehensive actions that can be adapted to local conditions.

Our study’s estimates of greater effects associated with NO_2_ was also reported in China for general emergency department visits as well as in Canada for visits for cardiovascular diseases [29,31]. Our study also found that the effects of different pollutants on visits for respiratory diseases differed by age group and were greater for the 5 to 9-year-old group. Similarly, a study by Alhanti et al. [30] of various cities in the United States reported that pollutants more greatly affected visits for asthma in the 5 to 18-year-old age group. In terms of cardiovascular diseases, our study identified adults over 65 years-old as the most affected group of adults, which is consistent with the ESCALA study’s findings on mortality [27]. These results make it possible to identify population groups that are more susceptible to the effects of air pollution, and thereby guide preventive actions and communications that are specifically aimed at these groups.

Since this is an ecological study, inferences can only be made at the population level and cannot be extrapolated to the individual level. Although confounding bias is likely given the ecological nature of this study, the estimates of the effects were adjusted for weather variables, which are the main confounders when determining these types of associations at the population level. Exposure misclassification is also a potential bias in this study as air pollutant’s exposure was assigned by taking average of air quality stations measurement within each city; however, it is possible that pollutant ‘s effect estimates were not affected as this exposure misclassification is probably non-differential across cities and if present it would bias the estimates toward the null effect. Another limitation of this study is the representativeness of the data for national inferences, given that the data analyzed corresponded to only four capital cities in Colombia, where criteria pollutant measurements were available. Although data about pollutants were available from registries in three other cities, the quality of those records and the data that they contained for this study’s time frame were not adequate for this analysis. Nonetheless, since the four cities analyzed were four of the five cities with the largest populations in Colombia, we believe that the national estimates are a good representation of the average effect on the Colombian population.

This study also had significant strengths which are worth mentioning. First, adjusted data was available from air quality monitoring networks in the cities, which complied with national and EPA guidelines for measuring criteria pollutants. Another strength is that the statistical analysis was performed using a time-series and a time-stratified conditional binomial regression, a method which has been proven to provide very robust estimations and requires fewer computations [17]. This particular study coupled these models with GEE or marginal models, which decreases bias related to poor model specifications and provides more robust estimates for making population inferences [20]. Lastly, given that the effect of each pollutant was estimated by age group, it was possible to determine heterogeneous effects and particular dose-response patterns by age group, thereby providing evidence of population groups that are more susceptible to the effects of air pollution.

## 5. Conclusions

This is the first multi-city study performed in Colombia and Latin America to determine the association between air pollution and respiratory and circulatory morbidity at the population level. The results confirm that associations exist between the concentration of air pollutants and respiratory and circulatory morbidity, including at concentrations below those stipulated by Colombian regulations and WHO quality guidelines. The study also found that the effects of air pollution differ among cities, and it identified age groups that are more susceptible to these effects.

## Figures and Tables

**Table 1:** Emergency department visits for respiratory, cardiovascular, and cerebrovascular diseases, by city and age group in Colombia, 2011–2014.

Diagnosis	Number of Emergency Department Visits (%)			
	Respiratory Diseases	Cardiovascular Diseases	Cerebrovascular Diseases	Total
**City**				
Bogotá	2,552,250 (75.8)	303,632 (64.6)	22,010 (49.4)	2,877,892 (74.2)
Bucaramanga	82,922 (2.5)	14,301 (3.0)	1795 (4.0)	99,019 (2.6)
Cali	288,062 (8.6)	61,430 (13.1)	9222 (20.7)	358,715 (9.2)
Medellín	441,753 (13.1)	90,490 (19.3)	11,512 (25.8)	543,756 (14.0)
Total	3,64,987 (100.0)	469,854 (100.0)	44,541 (100.0)	3,879,383 (100.0)
**Age group**				
0–4 years	326,520 (9.7)	242 (0.1)	42 (0.1)	326,804 (8.4)
5–9 years	961,192 (28.6)	1934 (0.4)	90 (0.2)	963,216 (24.8)
10–14 years	305,274 (9.1)	5530 (1.2)	70 (0.2)	310,874 (8.0)
15–44 years	1,103,541 (32.8)	133,167 (28.3)	3513 (7.9)	1,240,221 (32.0)
45–60 years	303,912 (9.0)	119,106 (25.3)	6275 (14.1)	429,293 (11.1)
>60 years	364,549 (10.8)	209,876 (44.7)	34,551 (77.6)	608,976 (15.7)
Total	3,364,988 (100.0)	469,854 (100.0)	44,541 (100.0)	3,879,383 (100.0)

**Table 2:** Daily Concentrations of Pollutants and Weather Conditions in Four Cities in Colombia, 2011 to 2014.

City/Variable	No. Days	Average	SD	P25	Median	P75
**Bogotá**						
CO (mg/m^3^)	1461	1.38	0.41	1.07	1.34	1.64
NO_2_ (µg/m^3^)	1460	32.34	8.04	26.65	31.69	37.52
SO_2_ (µg/m^3^)	1460	6.57	2.64	4.62	6.37	8.14
O_3_ (µg/m^3^)	1461	38.66	13.47	29.48	36.24	45.83
PM_10_ (µg/m^3^)	1460	49.80	15.54	38.00	48.47	60.39
PM_2.5_ (µg/m^3^)	1363	27.05	10.53	19.00	26.00	34.00
Temperature (°C)	1461	14.14	0.71	13.67	14.16	14.64
Relative humidity (%)	1461	64.58	5.02	61.15	64.11	68.17
Precipitation (mm)	1461	2.52	4.13	0.03	0.55	3.24
**Bucaramanga**						
CO (mg/m^3^)	1389	2.06	0.62	1.63	2.03	2.43
NO_2_ (µg/m^3^)	1455	23.48	11.83	12.4	22.4	32.17
SO_2_ (µg/m^3^)	871	10.50	4.07	7.25	10.10	13.40
O_3_ (µg/m^3^)	1454	48.31	13.92	38.75	46.55	55.87
PM_10_ (µg/m^3^)	1459	44.46	9.54	37.75	43.40	50.80
PM_2.5_ (µg/m^3^)	812	20.31	7.28	15.29	20.00	24.50
Temperature (°C)	1364	23.57	1.17	22.82	23.50	24.20
Relative humidity (%)	1137	59.20	25.22	37.18	72.28	77.89
Precipitation (mm)	1334	2.31	6.74	0.00	0.05	1.35
**Cali**						
CO (mg/m^3^)	607	1.74	0.58	1.40	1.75	2.20
NO_2_ (µg/m^3^)	441	14.45	4.53	11.30	14.60	17.30
SO_2_ (µg/m^3^)	721	19.45	26.74	6.90	10.50	16.50
O_3_ (µg/m^3^)	825	74.15	24.37	58.25	75.80	92.15
PM_10_ (µg/m^3^)	1263	32.42	12.79	23.51	32.55	41.50
PM_2.5_ (µg/m^3^)	637	15.71	4.49	12.50	15.50	18.50
Temperature (°C)	1385	24.84	1.34	23.95	25.06	25.84
Relative humidity (%)	1351	68.38	6.94	63.48	67.49	72.61
Precipitation (mm)	1390	7.32	17.88	0.00	0.00	4.82
**Medellín**						
CO (mg/m^3^)	712	2.69	1.05	1.97	2.47	3.10
NO_2_ (µg/m^3^)	982	37.83	9.93	31.30	36.90	43.63
SO_2_ (µg/m^3^)	0					
O_3_ (µg/m^3^)	842	56.47	16.49	44.65	55.25	67.53
PM_10_ (µg/m^3^)	1425	51.15	13.80	41.50	49.80	60.00
PM_2.5_ (µg/m^3^)	1437	26.01	7.91	20.00	25.20	30.66
Temperature (°C)	1364	23.56	1.17	22.82	23.50	24.20
Relative humidity (%)	1137	59.19	25.22	37.18	72.28	77.89
Precipitation (mm)	1333	2.32	6.74	0.00	0.05	1.35

**Table 3:** Estimates of the effect on the general population in terms of respiratory, cardiovascular, and cerebrovascular diseases, by type of pollutant and age group, Colombia, 2011–2014.

Pollutant	Age Group (Years)	Respiratory Diseases				Cardiovascular Diseases				Cerebrovascular Diseases				*n*
		Change *	95% CI		*p*-Vale	Change *	95% CI		*p*-Value	Change *	95% CI		*p*-Value	
**NO_2_** **(per increase in 6 μg/m^3^)**	**0–4**	**4.59**	2.91	6.31	0.000								0.000	61,360
	**5–9**	**10.59**	8.87	12.33	0.000									
	**10–14**	**4.81**	3.17	6.47	0.000									
	**15–44**	**2.25**	1.45	3.07	0.000	**0.96**	0.12	1.81	0.025	−0.06	−0.07	−0.05	0.000	
	**45–60**	−0.02	−0.03	−0.01	0.017	**2.56**	1.49	3.64	0.000	0.00	−0.02	0.48	0.236	
	**>60**	**2.62**	1.76	3.49	0.000	**6.12**	5.20	7.05	0.000	**6.17**	5.18	7.17	0.000	
**SO_2_** **(per increase in 2 μg/m^3^)**	**0–4**	−0.04	−0.05	−0.04	0.000									50,702
	**5–9**	**1.26**	0.77	1.76	0.000									
	**10–14**	0.64	0.15	1.14	0.010									
	**15–44**	0.54	0.32	0.75	0.000	0.21	−0.01	0.43	0.082	−0.01	−0.01	0.18	0.289	
	**45–60**	0.77	0.48	1.07	0.000	0.78	0.47	1.09	0.000	0.79	0.44	1.15	0.000	
	**>60**	1.09	0.85	1.32	0.000	0.79	0.55	1.04	0.000	0.58	0.33	0.83	0.000	
**PM_10_** **(per increase 10 μg/m^3^)**	**0–4**	**7.66**	5.90	9.45	0.000									82,043
	**5–9**	**8.32**	6.69	9.98	0.000									
	**10–14**	1.61	0.00	3.17	0.039									
	**15–44**	0.74	−0.01	1.50	0.051	0.96	0.17	1.76	0.017	−0.05	−0.06	−0.04	0.000	
	**45–60**	−0.05	−0.06	−0.04	0.000	1.41	0.40	2.43	0.006	−0.01	−0.02	0.38	0.198	
	**>60**	−0.03	−0.04	−0.03	0.000	**5.06**	4.19	5.93	0.000	**7.92**	6.99	8.86	0.000	
**PM_2.5_** **(per increase 5 μg/m^3^)**	**0–4**	−0.03	−0.04	−0.01	0.002									59,920
	**5–9**	**8.23**	6.62	9.86	0.000									
	**10–14**	**2.16**	0.64	3.7	0.005									
	**15–44**	−0.01	−0.01	0.59	0.732	−0.01	−0.01	0.74	0.998	−0.07	−0.08	−0.06	0.000	
	**45–60**	−0.03	−0.04	−0.02	0.000	**2.14**	1.15	3.13	0.000	−0.02	−0.03	0.00	0.006	
	**>60**	−0.01	−0.01	0.24	0.183	**5.61**	4.78	6.45	0.000	**7.17**	6.31	8.04	0.000	
**CO max 8 h** **(per increase in 0,4 mg/m^3^)**	**0–4**	0.29	0.19	0.40	0.000									61,360
	**5–9**	0.67	0.56	0.77	0.000									
	**10–14**	0.31	0.21	0.41	0.000									
	**15–44**	0.15	0.09	0.20	0.000	0.06	0.00	0.01	0.025	−0.01	−0.01	−0.01	0.000	
	**45–60**	−0.01	−0.01	0.00	0.017	0.17	0.09	0.23	0.000	−0.01	−0.01	0.03	0.236	
	**>60**	0.17	0.11	0.23	0.000	0.39	0.33	0.45	0.000	0.40	0.33	0.46	0.000	
**O3 max 8 h** **(per increase in 10 μg/m^3^)**	**0–4**	**4.64**	2.78	6.54	0.000									65,582
	**5–9**	0.58	−0.02	2.30	0.500									
	**10–14**	−0.02	−0.03	0.31	0.109									
	**15–44**	−0.01	−0.02	−0.01	0.040	1.31	0.04	0.21	0.002	1.26	0.03	2.51	0.044	
	**45–60**	0.76	−0.01	1.81	0.146	1.17	0.00	2.27	0.035	0.00	−0.02	0.79	0.453	
	**>60**	−0.01	−0.02	−0.01	0.028	0.41	−0.01	1.28	0.351	−0.01	−0.02	−0.01	0.042	

* Change refers to the percentage of change in the rate of emergency department visits for the specific disease groups, adjusted for temperature, relative humidity, precipitation, and their interactions with pollutant concentrations. All pollutants were centered to the value that represented 20% of the global mean concentration in the time-series for the four cities. Positive, statistically significant associations are shown in bold.

## Supplementary Materials

The following are available online at http://www.mdpi.com/1660-4601/15/8/1610/s1, Tables S1–S4: Estimates of the effect on the general population in terms of respiratory, cardiovascular, and cerebrovascular diseases, by type of pollutant and age group, 2011–2014, for each city.

## References

[1] Prüss-Üstün A., Wolf J., Corvalán C., Bos R., Neira M. (2016). Preventing Disease through Healthy Environments, a Global Assessment of the Burden of Disease from Environmental Risks.

[2] World Health Organization (2009). Country Profile of Environmental Burden of Disease—Colombia.

[3] Consejo Nacional de Política Económica y Social (2008). Documento CONPES 3550: Lineamientos Para la Formulacion de la Politica de Salud Ambiental con Enfasis en los Componentes de Calidad de Aire, Calidad de Agua y Seguridad Quimica. 3550.

[4] Rodriguez-Villamizar L.A., Gonzalez B.E., Vera L.M., Patz J., Bautista L.E. (2015). Environmental and occupational health research and training needs in Colombia: A Delphi study. Biomedica.

[5] Peñalosa R., Salamanca N., Rodríguez J.M., Rodríguez-García J.A.B. (2014). Burden of Disease Estimation for Colombia, 2010.

[6] Brunekreef B., Holgate S.T. (2002). Air pollution and health. Lancet.

[7] Pope C.A., Ezzati M., Dockery D.W. (2009). Fine-particulate air pollution and life expectancy in the United States. N. Engl. J. Med..

[8] Brook R.D. (2008). Cardiovascular effects of air pollution. Clin. Sci..

[9] Fajersztajn L., Saldiva P., Pereira L.A., Leite V.F., Buehler A.M. (2017). Short-term effects of fine particulate matter pollution on daily health events in Latin America: A systematic review and meta-analysis. Int. J. Public Health.

[10] Rodriguez-Villamizar L.A., Castro-Ortiz H., Rey-Serrano J.J. (2012). The effects of air pollution on respiratory health in susceptible populations: A multilevel study in Bucaramanga, Colombia. Cad. Saude Publica.

[11] Quiroz-Arcentales L., Hernandez-Florez L.J., Agudelo Calderon C.A., Medina K., Robledo-Martinez R., Osorio-Garcia S.D. (2013). PM10 exposure-related respiratory symptoms and disease in children living in and near five coal-mining areas in the Cesar department of Colombia. Rev. Salud Publica.

[12] Sarmiento R., Hernandez L.J., Medina E.K., Rodriguez N., Reyes J. (2015). Respiratory symptoms associated with air pollution in five localities of Bogota, 2008–2011, a dynamic cohort study. Biomedica.

[13] Blanco-Becerra L.C., Miranda-Soberanis V., Barraza-Villarreal A., Junger W., Hurtado-Diaz M., Romieu I. (2014). Effect of socioeconomic status on the association between air pollution and mortality in Bogota, Colombia. Salud Publica Mex..

[14] Blanco-Becerra L.C., Miranda-Soberanis V., Hernandez-Cadena L., Barraza-Villarreal A., Junger W., Hurtado-Diaz M., Romieu I. (2014). Effect of particulate matter less than 10microm (PM10) on mortality in Bogota, Colombia: A time-series analysis, 1998–2006. Salud Publica Mex..

[15] Bogota D.C., Republica de Colombia Ministerio de Salud y Proteccion Social (2012). Viability and Factibility of Using RIPS as Information Source for Public Health Surveillance.

[16] Proyecciones de Poblacion Colombia. https://www.dane.gov.co/index.php/estadisticas-por-tema/demografia-y-poblacion/proyecciones-de-poblacion.

[17] Armstrong B.G., Gasparrini A., Tobias A. (2014). Conditional Poisson models: A flexible alternative to conditional logistic case cross-over analysis. BMC Med. Res. Methodol..

[18] Cochran W. (1954). Some methods of strengthening the X2 goodness of fit test. Biometrics.

[19] Böhning D. (1994). A note on a test for Poisson overdispersion. Biometrika.

[20] Hubbard A.E., Ahern J., Fleischer N.L., Van der Laan M., Lippman S.A., Jewell N., Bruckner T., Satariano W.A. (2010). To GEE or not to GEE: Comparing population average and mixed models for estimating the associations between neighborhood risk factors and health. Epidemiology.

[21] Resolucion 2254 of 2017: Air Quality Standards for Colombia. http://www.minambiente.gov.co/images/normativa/app/resoluciones/96-res%202254%20de%202017.pdf.

[22] World Health Organization (2005). WHO Air Quality Guidelines for Particulate Matter, Ozone, Nitrogen Dioxide and Sulfur Dioxide.

[23] Katsouyanni K., Touloumi G., Samoli E., Al E. (2001). Counfounding and effect modification in the short-term effects of ambient particles on total mortality: Results from 29 European cities within the APHEA2 project. Epidemiology.

[24] Pope C.A., Burnett R.T., Thun M.J., Calle E.E., Krewski D., Ito K., Thurston G.D. (2002). Lung cancer, cardiopulmonary mortality, and long-term exposure to fine particulate air pollution. JAMA.

[25] Atkinson R.W., Kang S., Anderson H.R., Mills I.C., Walton H.A. (2014). Epidemiological time series studies of PM2.5 and daily mortality and hospital admissions: A systematic review and meta-analysis. Thorax.

[26] Adar S.D., Filigrana P.A., Clements N., Peel J.L. (2014). Ambient Coarse Particulate Matter and Human Health: A Systematic Review and Meta-Analysis. Curr. Environ. Health Rep..

[27] Romieu I., Gouveia N., Cifuentes L.A., de Leon A.P., Junger W., Vera J., Strappa V., Hurtado-Diaz M., Miranda-Soberanis V., Rojas-Bracho L. (2012). Multicity study of air pollution and mortality in Latin America (the ESCALA study). Res. Rep. Health Eff. Inst..

[28] Bell M.L., Samet J.M., Dominici F. (2004). Time-series studies of particulate matter. Annu. Rev. Public Health.

[29] Stieb D.M., Szyszkowicz M., Rowe B.H., Leech J.A. (2009). Air pollution and emergency department visits for cardiac and respiratory conditions: A multi-city time-series analysis. Environ. Health Glob. Access Sci. Source.

[30] Alhanti B.A., Chang H.H., Winquist A., Mulholland J.A., Darrow L.A., Sarnat S.E. (2016). Ambient air pollution and emergency department visits for asthma: A multi-city assessment of effect modification by age. J. Expo. Sci. Environ. Epidemiol..

[31] Chen G., Zhang Y., Zhang W., Li S., Williams G., Marks G.B., Jalaludin B., Abramson M.J., Luo F., Yang D. (2017). Attributable risks of emergency hospital visits due to air pollutants in China: A multi-city study. Environ. Pollut..

